# Parity and Risk of Colorectal Cancer: A Dose-Response Meta-Analysis of Prospective Studies

**DOI:** 10.1371/journal.pone.0075279

**Published:** 2013-09-30

**Authors:** Hong-Bo Guan, Qi-Jun Wu, Ting-Ting Gong, Bei Lin, Yong-Lai Wang, Cai-Xia Liu

**Affiliations:** 1 Department of Obstetrics and Gynecology, Shengjing Hospital, China Medical University, Shenyang, China; 2 Department of Epidemiology, Shanghai Cancer Institute, Renji Hospital, Shanghai Jiaotong University School of Medicine, Shanghai, China; 3 State Key Laboratory of Oncogene and Related Genes, Shanghai Cancer Institute, Renji Hospital, Shanghai Jiaotong University School of Medicine, Shanghai, China; University of Illinois at Chicago, United States of America

## Abstract

**Background:**

Association between parity and colorectal cancer (CRC) risk has been investigated by several epidemiological studies but results are controversial, yet a comprehensive and quantitative assessment of this association has not been reported so far.

**Methods:**

Relevant published studies of parity and CRC were identified using MEDLINE, EMBASE and Web of Science databases through end of April 2013. Two authors independently assessed eligibility and extracted data. Eleven prospective studies reported relative risk (RR) estimates and 95% confidence intervals (CIs) of CRC risk associated with parity. We pooled the RR from individual studies using fixed- or random-effects models and carried out heterogeneity and publication bias analyses.

**Results:**

The summary RR for the ever parity vs. nulliparous was 0.95 (95% CI: 0.88–1.02), with no heterogeneity (*Q* = 9.04, *P* = 0.443, *I* ^2^ = 0.5%). Likewise, no significant association was yielded for the highest vs. lowest parity number (RR = 1.02, 95% CI: 0.89–1.17), with moderate heterogeneity (*Q* = 17.48, *P* = 0.094, *I* ^2^ = 37.1%). Dose-response analysis still indicated no effect of parity on CRC risk and the summary RR of per one livebirth was 0.99 (95% CI: 0.96–1.02), with moderate of heterogeneity (*Q* = 16.50, *P*<0.021, *I* ^2^ = 57.6%). Similar results were observed among all the subgroup analyses. No evidence of publication bias and significant heterogeneity between subgroups were detected by meta-regression analyses.

**Conclusion:**

Results of this dose-response meta-analysis of prospective studies found that there was little evidence of an association between parity and CRC risk.

## Introduction

Colorectal cancer (CRC) is the second most commonly diagnosed cancer and third leading cause of cancer death worldwide in females, with over 570,100 new cases and 288,100 cancer deaths in 2008, which constituted a significant proportion of the global burden of cancer morbidity and mortality [1]. Primary prevention of CRC is therefore a major public health priority. Epidemiological studies suggested some modifiable risk factors for CRC including smoking, physical inactivity, overweight and obesity, red and processed meat consumption, and excessive alcohol consumption [2], [3]. Studies have also provided evidence that sex hormones, especially estrogen, might play a role in CRC pathogenesis [4]. Estrogen has been implicated for this association through several mechanisms that might involve reduction of secondary bile acid production, reduction of circulating insulin-like growth factor-I (IGF-I), and inhibiting cell proliferation of colorectal tumors by binding to the estrogen receptor [4], [5].

Reproductive factors, such as pregnancy, age at menarche, and age at menopause, have been used as surrogate markers for lifetime exposure to endogenous estrogens [6]. Estradiol and estriol are produced by the placenta, and maternal levels continue to increase over the course of the pregnancy [7]. Changes in maternal hormones during pregnancy might lead to etiological changes that affect CRC risk [8]. Several case-control studies have reported an inverse association between ever parity or parity number and CRC risk [9], [10], [11], [12]. However, the interpretation of traditional case-control studies is hampered by possible recall and a selection bias, even parity is likely less prone to recall bias and misclassification, which make it difficult to draw firm conclusions. Over the past decade, findings from prospective studies which have examined the association between parity and the risk of CRC have been inconsistent. Some studies found no association [13], [14], [15], whereas others reported a positive association with ever parity or higher parity numbers [16], [17]. The aim of this study was to clarify the relationship between parity and CRC risk by summarizing the evidence of published prospective studies with a dose-response meta-analysis.

## Materials and Methods

### Literature Search

We performed a comprehensively literature search to April 2013 using MEDLINE, EMBASE, and Web of Science databases for epidemiological studies evaluating the association between parity (defined as the total number of live-births) and the risk of CRC. The search was limited to studies of humans using the following search key words and medical subject heading terms: (parity OR pregnancy OR livebirth OR reproductive OR reproduction OR reproductive factors) AND (colorectal OR colorectum OR colon OR rectal OR rectum) AND (cancer OR neoplasm OR carcinoma OR tumor). We also reviewed the references of all included studies for additional publications. This systematic review was planned, conducted, and reported in adherence to standards of quality for reporting meta-analyses [18].

### Study Selection Criteria

**Figure 1:**
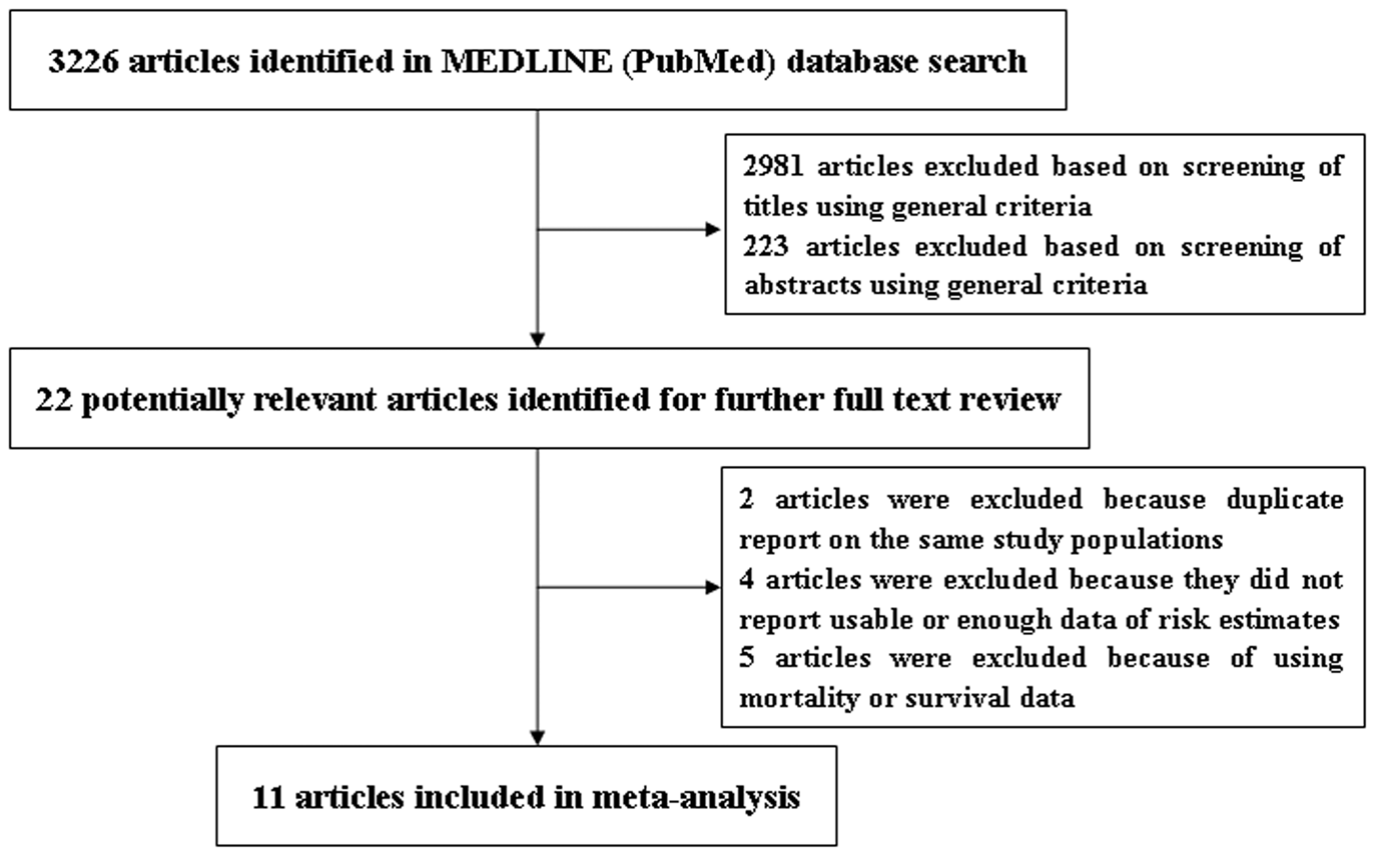
Selection of studies for inclusion in meta-analysis.

Published studies were included if they 1) used a prospective study design; 2) evaluated the association between parity and CRC risk; 3) presented relative risk (RR) or hazard ratio (HR) estimates with 95% confidence intervals (CI), standard errors (SE) or data necessary to calculate these. When multiple publications from the same study were available, we used the publication with the largest number of cases and most applicable information. The detailed steps of our literature search are shown in Figure 1. Briefly, we identified 22 potentially relevant full text publications [13], [14], [15], [16], [17], [19], [20], [21], [22], [23], [24], [25], [26], [27], [28], [29], [30], [31], [32], [33], [34], [35] from 3,226 articles. Two publications [16], [21] that did not report enough information for the main analysis of ever parity, thus they were just included in the subgroup analysis of the number of parity. Two articles were excluded because of duplicate reports from the same study populations [25], [26], four articles were excluded because they did not report usable or enough data of risk estimates [27], [28], [29], [30], and five articles were excluded because of using mortality or survival data [31], [32], [33], [34], [35]. The remaining 11 articles were included in the meta-analysis [13], [14], [15], [16], [17], [19], [20], [21], [22], [23], [24].

### Data Abstraction and Quality Assessment

For each eligible study, two investigators (H-BG and Q-JW) independently performed the eligibility evaluation, data abstraction, and quality assessment. The disagreements were discussed and resolved by consensus. Data abstracted from each study were: author list, year of publication, study region, study sample size (number of cases and cohort size), range of follow-up of studies, exposure and outcome assessment including parity and the number of parity categories, study-specific adjusted estimates with their 95% CIs for the ever parous versus nulliparous, highest versus lowest number (including nulliparous) of parity, and factors matched by in the design or adjusted for in data analysis. If multiple estimates of the association were available, we abstracted the estimate that adjusted for the most covariates. If no adjusted estimates were presented, we included the crude estimate. If no estimate was presented in a given study, we calculated it and its 95% CI according to the raw data presented in the article.

To assess study quality, a 9-star system on the basis of the Newcastle-Ottawa Scale [36], [37], [38] was used. A full score was 9 and a high quality study was defined as one with a quality score greater than or equal to 8.

### Statistical Analysis

The study-specific adjusted RRs were used as the measure of association across studies. Because the absolute risk of CRC is low, we assumed that estimates of risk, rate or hazard ratios from prospective studies were all valid estimates of the RR and we therefore report all results as the RR for simplicity. For one study that did not use the category with the lowest number of parity as the reference, we used the effective count method proposed by Hamling et al [39] to recalculate the RRs. For studies that reported separately on colon and rectal cancer, but not for colorectal cancer, we just pooled the separate results with other studies.

For the dose-response analysis, we used the method proposed by Greenland et al [40] and Orsini et al [41] to compute study-specific slopes (linear trends) and 95% CIs from the natural logs of the RRs and CIs across categories of the number of parity. The method requires that the distribution of cases and person-years or non-cases and the RRs with the variance estimates for at least three quantitative exposure categories are known. For studies that reported the number by ranges we estimated the midpoint in each category by calculating the average of the lower and upper bound. When the highest category was open ended we assumed the length of the open ended interval to be the same as that of the adjacent interval. When the lowest category was open ended we set the lower boundary to zero. The dose-response results in the forest plots are presented for a one livebirth increment for the number of parity.

We evaluated heterogeneity of RRs across studies by using the Cochrane *Q* statistic, where *P*<0.1 was indicative of statistically significant heterogeneity, and the *I* ^2^ statistic. The summary estimate based on the fixed-effects model [42] for no detected heterogeneity or the random-effects model [43] when substantial heterogeneity was detected. In both methods, the weight of each study depended on the inverse of the variance of log OR, which was estimated by the 95% CI from each study. Summary estimates were calculated for ever parous and the number of parity. Subgroup analyses were carried out based on study quality (low vs. high quality), duration of follow-up (<10 vs. ≥10 years), number of cases (<500 vs. ≥500), geographic location (America, Europe, and Asia), anatomic cancer site (colon vs. rectum), subsite of colon cancer (proximal vs. distal). We also stratified the included studies by whether the study adjusted for potentially important confounders and risk factors (e.g., body mass index, diabetes mellitus (DM), and physical activity). We do not stratify by case assessment because all included studies used cancer registries or medical records. Heterogeneity between subgroups was evaluated by meta-regression. Finally, we carried out sensitivity analyses excluding one study at a time to explore whether the results were strongly influenced by a specific study.

Publication bias was evaluated via Egger’s linear regression [44], Begg’s rank correlation methods [45] and funnel plots. A *P*-value less than 0.05 for Egger’s or Begg’s tests was considered representative of significant statistical publication bias. Statistical analyses were performed with Stata (version 11.2; StataCorp, College Station, TX). P-values were two sided with a significance level of 0.05.

## Results

### Study Characteristics and Quality Assessment

Table 1 represents the characteristics of the 11 included studies. Ten cohort [13], [14], [15], [16], [17], [19], [20], [21], [22], [24] and 1 nested case-control studies [23] were published between 1987 and 2011, which involved a total of 9,178 cases and 964,050 non-cases. Six studies were conducted in the United States [13], [15], [16], [17], [21], [24], 2 each in Europe [14], [23] and Japan [20], [22], and 1 in Canada [19]. Cohort sizes ranged from 11,888 [24] to 337,802 [14], and the number of CRC cases varied from 68 [24] to 2,148 [23]. The median number of CRC cases was 501 and median follow-up were 10 years.

**Table 1:** Characteristics of studies of parity and colorectal cancer risk.

First author, publication year (reference), Country, Study design	Cases/subject (age), duration of follow up	Parity categories (exposure/case assessment)	HR/RR (95% CI)	Matched/Adjusted factors
Zervoudakis et al [13], 2011, USA, CS	2,014/214,162 (50–71 y), 8.2 y	CRC Ever parous vs. Nulliparous	0.98 (0.86–1.12)	Age, BMI, education level, alcohol consumption, family history of colorectal cancer, race, smoking history, DM, PA, and use of hormone therapy
		CRC≥5 vs. Nulliparous	0.95 (0.79–1.14)	
		(Self-questionnaire/cancer registry)		
Tsilidis et al [14], 2010, European, CS	1,878/337,802 (35–70 y), 9 y	CRC Ever parous vs. Nulliparous	0.96 (0.83–1.10)	Participating center, age at recruitment, smoking status, DM, BMI, PA, and alcohol use
		CRC≥4 vs. 1	1.17 (0.97–1.42)	
		(Self-questionnaire/cancer registry)		
Akhter et al [20], 2008, Japan, CS	538/48,511 (40–69 y), 12 y	CRC Ever parous vs. Nulliparous	0.86 (0.60–1.24)	Age, PHC area, family history of colorectal cancer, BMI, leisure time PA, cigarette smoking, and alcohol drinking
		CRC≥3 vs. Nulliparous	0.83 (0.57–1.19)	
		CC Ever parous vs. Nulliparous	0.94 (0.59–1.49)	
		CC≥3 vs. Nulliparous	0.92 (0.57–1.47)	
		Proximal CC Ever parous vs. Nulliparous	0.83 (0.45–1.54)	
		Proximal CC≥3 vs. Nulliparous	0.87 (0.47–1.62)	
		Distal CC Ever parous vs. Nulliparous	1.09 (0.50–2.35)	
		Distal CC≥3 vs. Nulliparous	0.99 (0.45–2.14)	
		RC Ever parous vs. Nulliparous	0.71 (0.40–1.27)	
		RC≥3 vs. Nulliparous	0.70 (0.39–1.25)	
		(Self-questionnaire/cancer registry)		
Kabat et al [19], 2008, Canada, CS	1,142/89,835 (40–59 y), 16.4 y	CRC Ever parous vs. Nulliparous	1.19 (0.85–1.66)	Age, BMI, menopausal status, pack-years of smoking, OC use, HRT use, education, age at menarche, and age at first live birth
		CRC≥5 vs. Nulliparous	1.18 (0.81–1.71)	
		CC≥5 vs. Nulliparous	1.20 (0.77–1.86)	
		Proximal CC≥5 vs. Nulliparous	1.79 (0.94–3.39)	
		Distal CC≥5 vs. Nulliparous	0.79 (0.40–1.59)	
		RC≥5 vs. Nulliparous	1.05 (0.54–2.05)	
		(Self-questionnaire/cancer registry)		
Lin et al [21], 2007, USA, CS	267/39,680 (≥45 y), 11 y	CRC≥5 vs. Nulliparous	0.84 (0.53–1.35)	Age, randomized treatment assignment, family history of colorectal cancer, previous history of benign colorectal polyps, BMI, PA, smoking status, red meat intake, alcohol consumption, baseline aspirin use, multivitamin use, baseline postmenopausal hormone use, and OC use
		CC≥5 vs. Nulliparous	0.79 (0.45–2.37)	
		RC≥5 vs. Nulliparous	0.90 (0.33–2.47)	
		(Self-questionnaire/medical records)		
Tamakoshi et al [22], 2004,Japan, CS	207/38,420 (40–79 y), 7.6 y	CC Ever parous vs. Nulliparous	0.65 (0.35–1.20)	Age at baseline, study area, smoking status, alcohol drinking habit, exercise, meat intake, green leafy vegetable intake, family history of colon cancer, and BMI at baseline
		CC≥4 vs. 1	0.90 (0.47–1.74)	
		(Self-questionnaire/cancer registry)		
Troisi et al [15], 1997, USA, CS	203/57,529 (31–90 y), 10 y	CRC Ever parous vs. Nulliparous	0.87 (0.66–1.16)	Age
		CRC≥4 vs. Nulliparous	1.00 (0.72–1.50)	
		CC Ever parous vs. Nulliparous	0.87 (0.58–1.36)	
		CC≥4 vs. Nulliparous	0.91 (0.54–1.50)	
		Proximal CC Ever parous vs. Nulliparous	0.74 (0.42–1.31)	
		Proximal CC≥4 vs. Nulliparous	0.67 (0.31–1.40)	
		Distal CC Ever parous vs. Nulliparous	1.45 (0.65–3.23)	
		Distal CC≥4 vs. Nulliparous	1.80 (0.74–4.50)	
		RC Ever parous vs. Nulliparous	1.40 (0.58–3.39)	
		RC≥4 vs. Nulliparous	1.60 (0.57–4.30)	
		(Self-questionnaire/medical records)		
Martínez et al [16], 1997, USA, CS	501/89,448 (30–55 y), 12 y	CRC≥5 vs. 1	1.57 (1.02–2.41)	Age, BMI, PA, family history of colorectal cancer, aspirin use, cigarette smoking, alcohol consumption, intake of red meat, OC use, postmenopausal hormone use, age at menarche, age at first pregnancy, and age at menopause
		CC≥5 vs. 1	1.57 (0.97–2.53)	
		RC≥5 vs. 1	1.63 (0.63–4.21)	
		(Self-questionnaire/medical records)		
Broeders et al [23], 1996, Sweden, NC-CS	2,148/10,738 (20–59 y), 25 y	CC Ever parous vs. Nulliparous	0.90 (0.77–1.06)	Age
		CC≥5 vs. Nulliparous	0.77 (0.54–1.10)	
		Proximal CC Ever parous vs. Nulliparous	0.91 (0.68–1.22)	
		Proximal CC≥5 vs. Nulliparous	0.76 (0.38–1.52)	
		Distal CC Ever parous vs. Nulliparous	0.90 (0.71–1.15)	
		Distal CC≥5 vs. Nulliparous	0.74 (0.44–1.26)	
		RC Ever parous vs. Nulliparous	0.86 (0.69–1.06)	
		RC≥5 vs. Nulliparous	0.99 (0.64–1.51)	
		(NA/cancer registry)		
Bostick et al [17], 1994, USA, CS	212/35,215 (55–69 y), 5 y	CC Ever parous vs. Nulliparous	1.63 (0.93–2.86)	Age, total energy intake, height, total vitamin E intake, a total vitamin E by age interaction term, and vitamin A supplement intake
		CC≥3 vs. Nulliparous	1.80 (1.02–3.19)	
		(Self-questionnaire/cancer registry)		
Wu et al [24], 1987, USA, CS	68/11,888 (N/A), 4.5 y	CRC Ever parous vs. Nulliparous	1.05 (0.63–1.76)	None
		CRC≥3 vs. Nulliparous	0.50 (0.20–1.30)	
		(Self-questionnaire/medical records)		

HR: hazards ratio; RR: relative risk; CRC: colorectal cancer; CC: colon cancer; RC: rectal cancer; CI: confidence interval; NC-CS: nested case-control study; CS: cohort study; N/A: not available; BMI: body mass index; OC: oral contraceptive; PA: physical activity; DM: diabetes mellitus.

Study-specific quality scores are summarized in Table S1. The quality scores ranged from 6 to 9 with a median score of 8. Studies with a lower quality score generally did not adjust for any confounders. The high-quality studies (i.e., those studies that had at least a score of 8) included seven cohort studies [13], [14], [16], [19], [20], [21], [22].

### Ever vs. Never Parity

**Figure 2:**
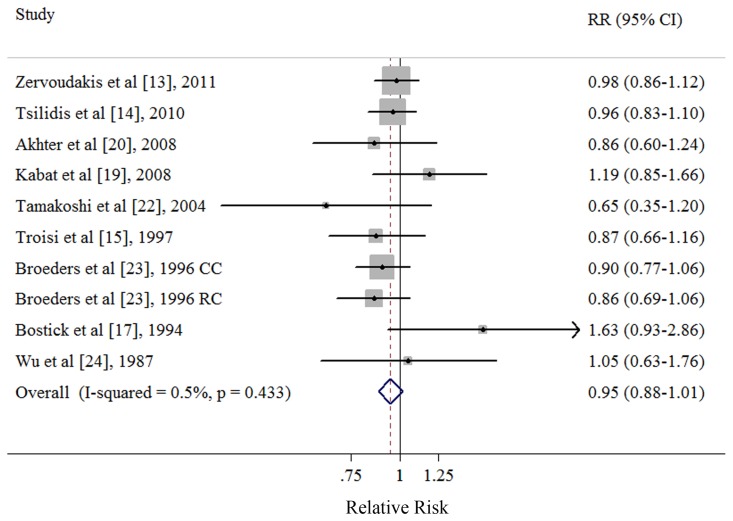
Forest plot (fixed-effects model) of ever parity and colorectal cancer risk. Squares indicate study-specific relative risks (size of the square reflects the study-specific statistical weight); horizontal lines indicate 95% CIs; diamond indicates the summary relative risk estimate with its 95% CI. CI: confidence interval; RR: relative risk; CC: colon cancer; RC: rectal cancer.

Eight cohort [13], [14], [15], [17], [19], [20], [22], [24] and 1 nested case-control studies [23] investigated the association between ever parity and CRC risk. The summary RR of CRC for the ever parity versus nulliparous was 0.95 (95% CI: 0.88–1.02), with no heterogeneity (*Q* = 9.04, *P* = 0.443, *I* ^2^ = 0.5%) (Table 2 and Figure 2). There was no indication of publication bias with Egger’s test (*P* for bias = 0.739) or with Begg’s test (*P* for bias = 0.929) and no asymmetry was observed in the funnel plots when inspected visually (data not shown).

**Table 2:** Summary risk estimates of the association between parity and colorectal cancer risk.

	No. of studies	Summary RR (95% CIs)	*Q* Statistic	*I* ^2^ Value (%)	*P*_h_ *	*P*_h_ **
**All studies**	9	0.95 (0.88–1.02)	9.04	0.5	0.433	–
**Subgroup analyses**						0.434
High quality studies (scores ≥8)	5	0.97 (0.89–1.06)	3.52	0	0.475	
Low quality studies (scores <8)	4	0.91 (0.81–1.02)	4.82	16.9	0.307	
Duration of follow-up						0.326
<10 y	5	0.98 (0.89–1.07)	5.01	20.1	0.287	
≥10 y	4	0.91 (0.82–1.01)	2.94	0	0.568	
Number of cases						0.980
<500	4	0.95 (0.77–1.17)	5.53	45.7	0.137	
≥500	5	0.95 (0.88–1.02)	3.52	0	0.621	
Geographic location						0.146
America	5	1.00 (0.90–1.12)	5	20	0.287	
Europe	2	0.92 (0.84–1.01)	0.80	0	0.670	
Asia	2	0.80 (0.59–1.09)	0.59	0	0.443	
Anatomic cancer site						0.650
Colon	5	0.92 (0.80–1.05)	5.35	25.2	0.253	
Rectum	3	0.86 (0.71–1.05)	1.59	0	0.451	
Cancer subsite of colon						0.611
Proximal colon	3	0.87 (0.68–1.10)	0.42	0	0.810	
Distal colon	3	0.95 (0.76–1.18)	1.38	0	0.501	
**Adjustment for confounders or important risk factors**						
Body mass index						0.434
Yes	5	0.97 (0.89–1.06)	3.52	0	0.475	
No	4	0.91 (0.82–1.02)	4.82	16.9	0.307	
Diabetes mellitus						0.498
Yes	2	0.97 (0.88–1.07)	0.04	0	0.834	
No	7	0.92 (0.83–1.02)	8.46	17.3	0.293	
Physical activity						0.808
Yes	4	0.95 (0.87–1.05)	1.97	0	0.579	
No	5	0.94 (0.84–1.04)	7.00	28.6	0.220	
Cigarette smoking						0.434
Yes	5	0.97 (0.89–1.06)	3.52	0	0.475	
No	4	0.91 (0.82–1.02)	4.82	16.9	0.307	
Alcohol drinking						0.808
Yes	4	0.95 (0.87–1.05)	1.97	0	0.579	
No	5	0.94 (0.84–1.04)	7.00	28.6	0.220	
HRT use						0.274
Yes	2	1.01 (0.89–1.14)	1.12	10.6	0.290	
No	7	0.92 (0.85–1.00)	6.55	0	0.478	
Family history of colorectal cancer/adenomatous polyposis						0.944
Yes	3	0.95 (0.84–1.07)	1.96	0	0.375	
No	6	0.95 (0.87–1.03)	7.08	15.3	0.314	

RR: relative risk; CI: confidence interval; HRT: hormone replacement therapy.

* *P* value for heterogeneity within each subgroup.

** *P* value for heterogeneity between subgroups with meta-regression analysis.

### Highest vs. Lowest Number of Parity

**Figure 3:**
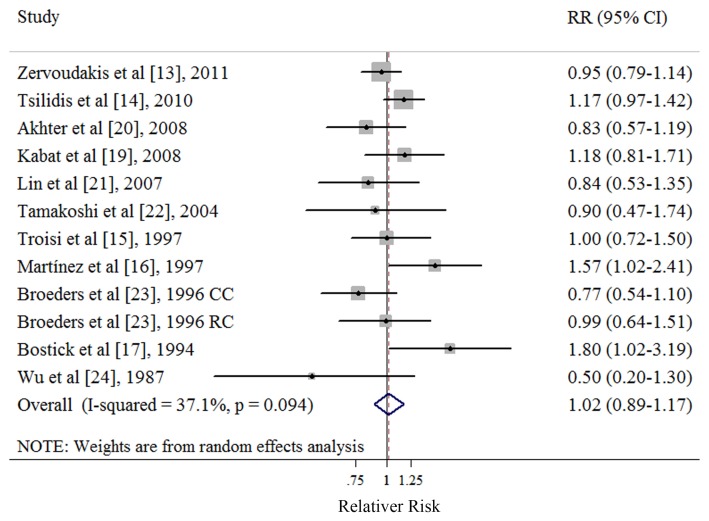
Forest plot (random-effects model) of parity number and colorectal cancer risk. Squares indicate study-specific relative risks (size of the square reflects the study-specific statistical weight); horizontal lines indicate 95% CIs; diamond indicates the summary relative risk estimate with its 95% CI. CI: confidence interval; RR: relative risk; CC: colon cancer; RC: rectal cancer.

Ten cohort [13], [14], [15], [16], [17], [19], [20], [21], [22], [24] and 1 nested case-control studies [23] investigated the association between the number of parity and CRC risk. Eight studies [13], [15], [17], [19], [20], [21], [23], [24] referred to nulliparous as the lowest category of parity number and 3 studies [14], [16], [22] referred to one livebirth as the lowest category of parity number. The summary RR of CRC for the highest versus lowest categories of the number of parity was 1.02 (95% CI: 0.89–1.17), with moderate heterogeneity (*Q* = 17.48, *P* = 0.094, *I* ^2^ = 37.1%) (Table 3 and Figure 3). There was no indication of publication bias with Egger’s test (*P* for bias = 0.734) or with Begg’s test (*P* for bias = 0.891) and no asymmetry was seen in the funnel plots when inspected visually (data not shown).

**Table 3:** Summary risk estimates of the association between the number of parity and colorectal cancer risk, highest vs. lowest parity number.

	No. of studies	Summary RR (95% CIs)	*Q* Statistic	*I* ^2^ Value (%)	*P*_h_ *	*P*_h_ **
**All studies**	11	1.02 (0.89–1.17)	17.48	37.1	0.094	–
**Subgroup analyses**						0.611
High quality studies (scores ≥8)	7	1.05 (0.94–1.17)	8.78	31.6	0.186	
Low quality studies (scores <8)	4	0.97 (0.72–1.31)	8.08	50.5	0.089	
Duration of follow-up						0.641
<10 y	5	1.06 (0.84–1.34)	8.43	52.6	0.077	
≥10 y	6	0.99 (0.85–1.14)	8.54	29.7	0.201	
Number of cases						0.847
<500	5	1.00 (0.79–1.26)	6.82	41.4	0.145	
≥500	6	1.03 (0.93–1.15)	10.59	43.4	0.102	
Geographic location						0.339
America	7	1.08 (0.88–1.34)	11.65	48.5	0.070	
Europe	2	1.06 (0.90–1.23)	4.23	52.7	0.121	
Asia	2	0.85 (0.61–1.17)	0.04	0	0.833	
Anatomic cancer site						0.977
Colon	8	1.04 (0.87–1.24)	10.67	34.4	0.154	
Rectum	6	1.04 (0.81–1.35)	4.57	0	0.470	
Cancer subsite of colon						0.881
Proximal colon	4	0.98 (0.70–1.36)	5.02	40.3	0.170	
Distal colon	4	0.90 (0.64–1.27)	2.99	0	0.393	
**Adjustment for confounders or important risk factors**						
Body mass index						0.611
Yes	7	1.05 (0.94–1.17)	8.78	31.6	0.186	
No	4	0.97 (0.72–1.31)	8.08	50.5	0.089	
Diabetes mellitus						0.787
Yes	2	1.05 (0.92–1.20)	2.38	58.0	0.123	
No	9	1.01 (0.84–1.21)	14.87	39.5	0.095	
Physical activity						0.886
Yes	6	1.04 (0.93–1.16)	8.36	40.2	0.138	
No	5	1.00 (0.84–1.20)	9.02	44.6	0.108	
Cigarette smoking						0.611
Yes	7	1.05 (0.94–1.17)	8.78	31.6	0.186	
No	4	0.97 (0.72–1.31)	8.08	50.5	0.089	
Alcohol drinking						0.886
Yes	6	1.04 (0.93–1.16)	8.36	40.2	0.138	
No	5	1.00 (0.84–1.20)	9.02	44.6	0.108	
HRT use						0.616
Yes	4	1.03 (0.89–1.19)	5.68	47.2	0.128	
No	7	1.03 (0.90–1.17)	11.80	40.7	0.107	
Family history of colorectal cancer/adenomatous polyposis						0.631
Yes	5	0.95 (0.84–1.07)	5.97	33.0	0.201	
No	6	0.95 (0.87–1.03)	10.38	42.2	0.110	

RR: relative risk; CI: confidence interval; HRT: hormone replacement therapy.

* *P* value for heterogeneity within each subgroup.

** *P* value for heterogeneity between subgroups with meta-regression analysis.

In a sensitivity analysis, we sequentially removed one study at a time and re-analyzed the data. The 11 study-specific RRs of the number of parity ranged from a low of 1.00 (95% CI: 0.91–1.11, *Q* = 13.54, *P* = 0.196, *I* ^2^ = 26.1%) after omission of the study by Martínez et al [16] to a high of 1.05 (95% CI: 0.95–1.17, *Q* = 14.69, *P* = 0.100, *I* ^2^ = 38.7%) after omission of the study by Broeders et al [23]. The effect on the results of excluding three studies [14], [16], [22] which referred to one livebirth as the lowest category of parity number was also explored and the summary RR was 0.95 (95% CI: 0.85–1.07, *Q* = 10.16, *P* = 0.254, *I* ^2^ = 21.3%).

### Dose-response Analysis of per 1 Livebirth

**Figure 4:**
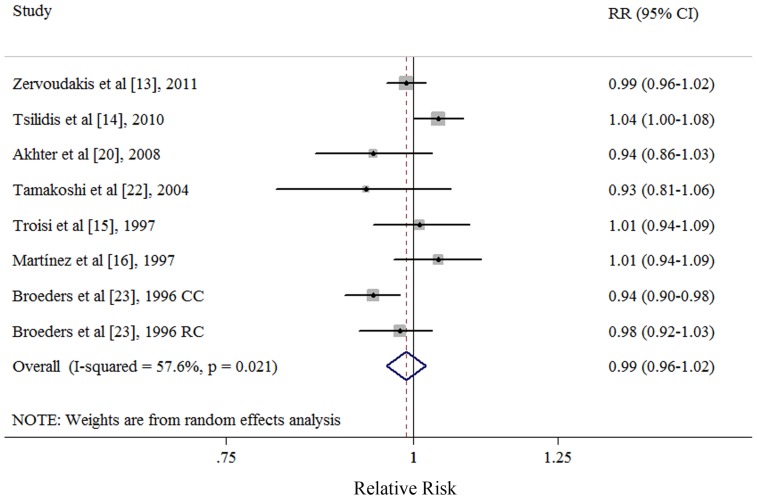
Dose-response analyses between per 1 livebirth and risk of colorectal cancer. Squares indicate study-specific relative risks (size of the square reflects the study-specific statistical weight); horizontal lines indicate 95% CIs; diamond indicates the summary relative risk estimate with its 95% CI. CI: confidence interval; RR: relative risk; CC: colon cancer; RC: rectal cancer.

Six cohort [13], [14], [15], [16], [20], [22] and 1 nested case-control studies [23] were included in the dose-response analysis. Four studies [13], [15], [20], [23] referred to nulliparous as the lowest category of parity number and 3 studies [14], [16], [22] referred to one livebirth as the lowest category of parity number. The summary RR of per livebirth was 0.99 (95% CI: 0.96–1.02), with moderate of heterogeneity (*Q* = 16.50, *P*<0.021, *I* ^2^ = 57.6%) (Figure 4). Publication bias was not evident with Egger’s test (*P* = 0.656), Begg’s test (*P* = 0.458) and visual inspect of the funnel plot (data not shown). Additionally, we found no evidence that the number of parity was associated with colon cancer, rectal cancer, and the subsite of colon cancer (Table 4).

**Table 4:** Summary risk estimates of the association between the number of parity and colorectal cancer risk, dose-response analysis of per 1 livebirth.

	No. of studies	Summary RR (95% CIs)	*Q* Statistic	*I* ^2^ Value (%)	*P*_h_ *	*P*_h_ **
**All studies**	7	0.99 (0.96–1.02)	16.50	57.9	0.021	–
**Subgroup analyses**						0.284
High quality studies (scores ≥8)	5	1.00 (0.97–1.04)	8.36	52.2	0.079	
Low quality studies (scores <8)	2	0.96 (0.93–1.00)	3.20	37.4	0.202	
Duration of follow-up						0.440
<10 y	3	1.01 (0.96–1.05)	5.25	61.9	0.072	
≥10 y	4	0.98 (0.94–1.02)	7.87	49.2	0.096	
Number of cases						0.910
<500	2	1.00 (0.79–1.26)	1.11	9.9	0.292	
≥500	5	0.99 (0.93–1.06)	15.39	67.5	0.009	
Geographic location						0.219
America	3	1.00 (0.97–1.03)	1.79	0	0.408	
Europe	2	0.99 (0.93–1.05)	12.11	83.5	0.002	
Asia	2	0.94 (0.87–1.01)	0.02	0	0.897	
Anatomic cancer site						0.814
Colon	4	0.99 (0.93–1.06)	9.11	67.1	0.028	
Rectum	4	0.98 (0.93–1.03)	2.58	0	0.462	
Cancer subsite of colon						0.922
Proximal colon	3	0.96 (0.90–1.02)	0.23	0	0.890	
Distal colon	3	0.99 (0.88–1.12)	4.90	59.2	0.086	
**Adjustment for confounders or important risk factors**						
Body mass index						0.284
Yes	5	1.00 (0.97–1.04)	8.36	52.2	0.079	
No	2	0.96 (0.93–1.00)	3.20	37.2	0.202	
Diabetes mellitus						0.266
Yes	2	1.01 (0.97–1.06)	3.89	74.3	0.049	
No	5	0.97 (0.95–1.00)	8.29	39.7	0.141	
Physical activity						0.284
Yes	5	1.00 (0.97–1.04)	8.36	52.2	0.079	
No	2	0.96 (0.93–1.00)	3.20	37.4	0.202	
Cigarette smoking						0.284
Yes	5	1.00 (0.97–1.04)	8.36	52.2	0.079	
No	2	0.96 (0.93–1.00)	3.20	37.4	0.202	
Alcohol drinking						0.284
Yes	5	1.00 (0.97–1.04)	8.36	52.2	0.079	
No	2	0.96 (0.93–1.00)	3.20	37.4	0.202	
HRT use						0.471
Yes	2	1.00 (0.97–1.03)	1.71	41.4	0.191	
No	5	0.98 (0.93–1.02)	14.45	65.4	0.013	
Family history of colorectal cancer/adenomatous polyposis						0.886
Yes	4	0.99 (0.97–1.02)	4.15	27.7	0.246	
No	3	0.99 (0.94–1.04)	12.33	75.7	0.006	

RR: relative risk; CI: confidence interval; HRT: hormone replacement therapy.

* *P* value for heterogeneity within each subgroup.

** *P* value for heterogeneity between subgroups with meta-regression analysis.

In a sensitivity analysis excluding one study at a time, the summary RR for CRC ranged from 0.98 (95% CI: 0.96–1.00, *Q* = 9.13, *P* = 0.166, *I* ^2^ = 34.3%) when Tsilidis et al [14] was excluded to 1.01 (95% CI: 0.99–1.03, *Q* = 8.37, *P* = 0.137, *I* ^2^ = 40.3%) when Broeders et al [23] was excluded. The effect on the results of excluding studies from the dose-response analysis was also explored. When the analysis of high versus low parity number was restricted to the studies that were included in the dose-response analysis of the number of parity, the summary RR was 1.02 (95% CI: 0.92–1.13, *Q* = 10.20, *P* = 0.177, *I* ^2^ = 31.4%), similar to the original analysis including all studies. Similarly, we also explored the effect on the results of excluding three studies which referred to one livebirth as the lowest category of parity number and the summary RR was 0.98 (95% CI: 0.96–1.00, *Q* = 5.34, *P* = 0.254, *I* ^2^ = 25.1%).

### Subgroup and Meta-regression Analyses

We carried out stratified and meta-regression analyses to examine possible differences between risk estimates by various study characteristics. However, we did not find evidence of heterogeneity and significant association between ever parity and the number of parity and CRC risk in pooled estimates by any subgroups analyses (Table 2 and 3). When considering about whether the included studies adjusted for potential important confounders or risk factors, we did not find a significant difference between estimates adjusted and those not adjusted for specific factors (Table 2 and 3). Similar results were also observed when the stratified analyses were carried out to the studies that were included in the dose-response analysis of the number of parity (Table 4).

## Discussion

To our knowledge, this is the first quantitative summary of the published literature investigated the relationship between parity and CRC risk. However, we found no evidence to support an association between ever parity and parity number and CRC risk in categorical and dose-response meta-analyses. In addition, the results were consistent in all the stratified analyses (Table 2, 3, and 4).

The exact biologic mechanisms underlying the association between parity and risk of CRC are not fully understood. However, to date, some biological evidence has suggested that there is a link between parity and CRC risk. Estrogens, which are commonly held that decreased transit time and increased bowel motility reduce risk by minimizing contact between lumen carcinogens and the colonic epithelium, or by limiting opportunity for activation of procarcinogens by epithelial metabolic enzymes, are 10-fold higher due to fetal-placental contribution during the pregnancy [46]. Furthermore, estrogen was also suggested to be involved in reduction of secondary bile acid production, circulating IGF-I, and inhibiting cell proliferation of colorectal tumors by binding to the estrogen receptor. On the other hand, hyperinsulinemia is a human CRC promoter based on evidence that insulin is a colon epithelial cell mitogen in vitro, and insulin delivered via injection was shown recently to increase the incidence of azoxymethane-initiated colon tumors in rats [47], [48]. And pregnancy disturbs carbohydrate metabolism leading to decreased glucose tolerance and increased secretion of insulin [46]. Although DM has already considered as a risk factors of CRC [49], limited number of the included studies [13], [14] adjusted it in their multivariable model. Even though the result of meta-regression of category and dose-response analysis did not suggest whether adjust DM is not the source of heterogeneity, further studies with adjustment for more confounding factors including DM are needed (Table 2, 3, and 4).

In the stratified analysis of the geographic location, though the results of meta-regression found no significant difference between the subgroups, the summary RR of Asia was slightly different from America and Europe not only in ever parity but in the analysis of the parity number (Table 2 and 3). Such a difference might be attributed to the different percentage of nulliparous populations in Asia than that found in America and Europe. Akhter et al [20] reported that about 6% non-cases did not give a live birth in a cohort study of 48,511 females conducted in Japan, whereas two prospective studies conducted in the United States [13] and Europe [14] reported almost 15% and 20.6% nulliparous populations in 212,148 and 335,924 non-cases, respectively. Similar differences were also observed when we compared the highest versus the lowest number of parity (Table 3). However, when the non-cases or person-years were considered in the dose-response analysis of parity number, the difference was attenuated (Table 4).

Our study has several strengths. Because the quantitative assessment was based on prospective studies, thus our findings are unlikely to be explained by recall bias and selection bias. We also carried out sensitivity and stratified analyses to investigate whether any particular study explained the results and explore the heterogeneity, but the findings were generally similar. Additionally, there was no evidence of significant heterogeneity between subgroups with meta-regression analyses. Although La Vecchia et al [50] have already reviewed published observational studies (including 15 case-control studies, two cohort studies, and one cancer registry-based study from seven different countries) which focused on the association between parity and CRC risk, the results of all included studies were just illustrated in their study. Compared to La Vecchia et al [50], this meta-analysis first comprehensively and quantitatively assessed this association to date and provided more detail information. Several limitations also should be addressed. First, this meta-analysis includes 11 good design prospective studies, but the possibility that the observed relation between parity and CRC risk was due to unmeasured or residual confounding should be considered. A number of factors may confound the association between CRC and reproductive variables, e.g., body mass index, DM, and socio-economic status. Although stratified analyses were carried out among these important confounders and risk factors and no difference was observed by meta-regression, several results showed borderline significance. Considering no access to the raw data from the included studies of this meta-analysis and we could not fully adjust for these potentially important confounders, thus, some of the unexplained between-study heterogeneity maybe attributed to the differences in statistical adjustments across studies and collaborative pooled studies which could standardize definitions of all the covariate categories across studies are warranted in the future. Second, this study does not provide a high level of evidence in the stratified analyses of anatomic CRC site and subsite of colon cancer though we involved a number of prospective studies, given the paucity of published studies. Therefore, further studies should evaluate this topic in the future. Thirdly, although publication bias can be a problem in meta-analyses of published studies, we found no evidence of such bias in this analysis. In addition, the studies that were excluded from the dose-response analysis of the number of parity are unlikely to have altered the results because the results of high versus low parity number were similar when we repeated the analyses with the same dataset as in the dose-response analysis.

In conclusion, this meta-analysis found that there was no association between ever parity and the number of parity and the risk of CRC not only in categorical and dose-response meta-analyses. More prospective studies or a collaborative re-analysis of primary data from the individual studies are warranted to provide more detailed results, including stratified results by anatomic CRC site, subsite of colon cancer, or adjustment for more potential confounders.

## Supporting Information

Table S1 Methodological quality of the prospective studies included in the meta-analysis.
(DOC)
