# Supplementary material for: Parity and Risk of Colorectal Cancer: A Dose-Response Meta-Analysis of Prospective Studies
Source: PLoS One. 2013 Sep 30;8(9):e75279. doi: 10.1371/journal.pone.0075279 (PMC3787107; doi:10.1371/journal.pone.0075279)
Supplement: Table S1 — Methodological quality of the prospective studies included in the meta-analysis. (DOC) [file pone.0075279.s001.doc]

**Table S1 Methodological quality of prospective studies included in the meta-analysis***

| First author,  publication year  [reference] | Representativeness  of the exposed cohort | Selection of the unexposed  cohort | Ascertainment  of exposure | Outcome of interest  not present  at start of study | Control for  important factor or additional factor^†^ | Assessment of outcome | Follow-up  long enough for outcomes  to occur^‡^ | Adequacy of  follow-up  of cohorts^§^ | Total quality  scores |
| --- | --- | --- | --- | --- | --- | --- | --- | --- | --- |
| Zervoudakis et al [13], 2011 | ⚝ | ⚝ | ⚝ | ⚝ | ⚝⚝ | ⚝ | — | ⚝ | 8 |
| Tsilidis et al [14], 2010 | ⚝ | ⚝ | ⚝ | ⚝ | ⚝⚝ | ⚝ | — | ⚝ | 8 |
| Akhter et al [20], 2008 | ⚝ | ⚝ | ⚝ | ⚝ | ⚝⚝ | ⚝ | ⚝ | ⚝ | 9 |
| Kabat et al [19], 2008 | ⚝ | ⚝ | ⚝ | ⚝ | ⚝ | ⚝ | ⚝ | ⚝ | 8 |
| Lin et al [21], 2007 | ⚝ | ⚝ | ⚝ | ⚝ | ⚝⚝ | ⚝ | ⚝ | ⚝ | 9 |
| Tamakoshi et al [22], 2004 | ⚝ | ⚝ | ⚝ | ⚝ | ⚝⚝ | ⚝ | — | ⚝ | 8 |
| Troisi et al [15], 1997 | ⚝ | ⚝ | ⚝ | ⚝ | — | ⚝ | — | ⚝ | 6 |
| Martínez et al [16], 1997 | ⚝ | ⚝ | ⚝ | ⚝ | ⚝⚝ | ⚝ | ⚝ | ⚝ | 9 |
| Broeders et al [23], 1996 | ⚝ | ⚝ | — | ⚝ | — | ⚝ | ⚝ | ⚝ | 6 |
| Bostick et al [17], 1994 | ⚝ | ⚝ | ⚝ | ⚝ | — | ⚝ | — | ⚝ | 6 |
| Wu et al [24], 1987 | ⚝ | ⚝ | ⚝ | ⚝ | — | ⚝ | — | ⚝ | 6 |

* A study could be awarded a maximum of one star for each item except for the item Control for important factor or additional factor. The definition/explanation of each column of the Newcastle-Ottawa Scale is available from (http://www.ohri.ca/programs/clinical_epidemiology/oxford.htm.).

† A maximum of 2 stars could be awarded for this item. Studies that controlled for body mass index received one star, whereas studies that controlled for other important confounders or risk factors such as diabetes mellitus, family history of colorectal cancer or adenomatous polyposis received an additional star.

‡ A cohort study with a follow-up time >10 y was assigned one star.

§ A cohort study with a follow-up rate >75% was assigned one star.
